# Effect of Modified Bioceramic Mineral Trioxide Aggregate Cement with Mesoporous Nanoparticles on Human Gingival Fibroblasts

**DOI:** 10.3390/cimb46040188

**Published:** 2024-03-30

**Authors:** Alexandra Kalash, Ioannis Tsamesidis, Georgia K. Pouroutzidou, Eleana Kontonasaki, Dimitrios Gkiliopoulos, Aristidis Arhakis, Konstantinos N. Arapostathis, Anna Theocharidou

**Affiliations:** 1 Department of Dentistry, School of Health Sciences, Aristotle University of Thessaloniki, GR-54124 Thessaloniki, Greece; alexandrakalash@outlook.com (A.K.); johntsame@gmail.com (I.T.); gpourout@physics.auth.gr (G.K.P.); kont@dent.auth.gr (E.K.); arhakis@dent.auth.gr (A.A.); koarap@dent.auth.gr (K.N.A.); 2 Laboratory of Chemical and Environmental Technology, Department of Chemistry, Aristotle University of Thessaloniki, GR-54124 Thessaloniki, Greece; dgiliopo@chem.auth.gr

**Keywords:** MTA, mesoporous silica nanoparticles, cerium doping, calcium doping, human gingival fibroblasts

## Abstract

The ion doping of mesoporous silica nanoparticles (MSNs) has played an important role in revolutionizing several materials applied in medicine and dentistry by enhancing their antibacterial and regenerative properties. Mineral trioxide aggregate (MTA) is a dental material widely used in vital pulp therapies with high success rates. The aim of this study was to investigate the effect of the modification of MTA with cerium (Ce)- or calcium (Ca)-doped MSNs on the biological behavior of human gingival fibroblasts (hGFs). MSNs were synthesized via sol–gel, doped with Ce and Ca ions, and mixed with MTA at three ratios each. Powder specimens were characterized using Fourier-transform infrared spectroscopy (FTIR), X-ray diffraction (XRD), and scanning electron microscopy (SEM). Biocompatibility was evaluated using a 3-[4,5-dimethylthiazol-2-yl]-2,5 diphenyl tetrazolium bromide (MTT) assay following hGFs’ incubation in serial dilutions of material eluates. Antioxidant status was evaluated using Cayman’s antioxidant assay after incubating hGFs with material disc specimens, and cell attachment following dehydration fixation was observed through SEM. Material characterization confirmed the presence of mesoporous structures. Biological behavior and antioxidant capacity were enhanced in all cases with a statistically significant increase in CeMTA 50.50. The application of modified MTA with cerium-doped MSNs offers a promising strategy for vital pulp therapies.

## 1. Introduction

The early loss of primary teeth can lead to functional and esthetic problems, as well as dental arch discrepancies in the permanent dentition; therefore, the preservation of primary teeth until their physiological exfoliation time is of high importance [1]. Pediatric endodontics includes the pulpal treatment of primary teeth and young permanent teeth in order to preserve them until the eruption of their permanent successors and ensure their continued root development and presence in the oral cavity, respectively [2]. Vital pulp therapy is a treatment modality widely applied in the field of pediatric dentistry for the treatment of carious primary and immature permanent teeth with normal pulp or showing signs of reversible pulpitis. Vital pulp therapies include indirect pulp treatment (IPT), direct pulp cap (DPC), and pulpotomy. Several agents such as calcium hydroxide, mineral trioxide aggregate (MTA), formocresol, and ferric sulfate have been used as medicaments in different vital pulp therapies [3]. MTA is an active biomaterial with calcium, silica, and bismuth as the main elemental components [4]. It has several desirable properties such as biocompatiblity, bioactivity, hydrophilicity, radiopacity, sealing ability, and low solubility, while its main drawbacks include discoloration, handling difficulties, long setting time, and high cost [5,6]. MTA has the potential to stimulate an ideal healing environment. When in direct contact with connective tissue, it forms calcium hydroxide, releasing calcium ions and thus aiding in cell attachment and proliferation. MTA modulates cytokine production, and its high pH provides antibacterial properties. It stimulates the differentiation and migration of hard tissue-producing cells and forms hydroxyapatite, providing a biological seal [7]. Since its introduction in 1993 by Torabinejad and White, MTA has undergone several modifications over the years in order to improve its properties [5].

The incorporation of nanotechnology into the field of medicine has gained much attention in recent decades [8]. Currently, of all the types of nanoparticles available, mesoporous silica nanoparticles (MSNs) are considered to be one of the most effective ones as they can additionally act as multifunctional delivery platforms [9]. MSNs are biocompatible inorganic nanocarriers that have desirable structural properties such as a large internal surface area and pore volume, tunable pore sizes (2–50 nm), and colloidal stability [8,10,11]. They possess a high loading capacity which is efficacious in drug encapsulation and site-specific release [12]. Based on their structural properties, MSNs are classified into five categories: molecular 41 sieves (M41S), Santa Barbara Amorphous (SBA), organically modified silica (ORMOSIL), hollow-type MSNs (HMSNs), and periodic mesoporous organosilica (PMO) [13]. Mobil Composition of Matter-41 (MCM-41), which belongs to the M41S category, is the most commonly used type of MSNs in biomedical applications [13,14]. It presents a hexagonal arrangement of mesopores with a pore size between 1.5 and 8 nm [15]. The incorporation of active ions such as Ca, Ce, Mg, and Cu into MSNs has been applied in an attempt to improve the properties of MSNs [16]. Cerium oxide is a rare earth metal which has received great interest due to its antioxidant, antimicrobial, anti-inflammatory, and regenerative properties [17,18,19]. In the field of medicine, several studies have found that cerium ions promote the differentiation and proliferation of osteoblasts and could have a great potential in treating bone diseases such as osteoporosis [18,20]. Loaded MSNs have also been applied in the field of dentistry. MSNs loaded with chlorhexidine added to dental materials (e.g., composites and glass ionomer cement) enhanced anti-biofilm properties while maintaining their mechanical properties [21,22]. Canto et al. reported that calcium-doped MSNs were more effective than casein phosphopeptide–amorphous calcium phosphate (CPP-ACP) and CPP-ACP/F mousse in preventing dental erosion [23]. Jun et al. demonstrated that cerium nanoparticles incorporated in MTA were able to enhance odontoblastic differentiation without affecting its mechanical properties [24]. To our knowledge, there are no studies examining the effect of loading MTA with calcium-doped and calcium/cerium co-doped MSNs (Ca-doped MSNs, Ca/Ce co-doped MSNs). Therefore, the aim of the present study was the synthesis, characterization, and the evaluation of the biological behavior of MTA loaded with cerium- and calcium-doped MSNs on human gingival fibroblasts (hGFs).

## 2. Materials and Methods

### 2.1. Material Synthesis

#### 2.1.1. Synthesis of Mesoporous Ca-Doped and Ca/Ce Co-Doped MSNs

Tetraethyl orthosilicate (TEOS, Sigma-Aldrich, St. Louis, MO, USA) was used as the silica source, and cetyltrimethylammonium bromide (CTAB, Sigma-Aldrich, St. Louis, MO, USA) as the structure-directing agent. Cerium(III) nitrate hexahydrate (Alfa Aesar, Haverhill, MA, USA) was used as the cerium ions’ source, while calcium nitrate tetrahydrate (ITW Reagents, Barcelona, Spain) was used as the calcium ions’ source.

The Ca-doped MSNs consisted of 60%mol SiO_2_ and 40%mol CaO, and the Ca/Ce co-doped MSNs consisted of 60%mol SiO_2_ 37.5%mol CaO and 2.5%CeO.

Calcium- and cerium-doped mesoporous silica nanoparticles (MCM-41 type) were synthesized in basic (pH 12–12.5) aqueous solution via the surfactant-assisted cooperative self-assembly process. A typical synthesis was conducted using two solutions. Solution 1 consisted of 2 g CTAB dissolved in 800 mL of aqueous NaOH 1.25% *w*/*v* at 80 °C. Solution 2 was prepared by dissolving 10 g of TEOS in 200 mL of deionized H_2_O at room temperature, followed by the addition of 8 g calcium nitrate tetrahydrate or 7.8 g calcium nitrate tetrahydrate and 0.28 g cerium nitrate hexahydrate to produce Ca-doped or Ca/Ce co-doped nanoparticles, respectively. Solution 2 was added to solution 1 dropwise, and the resulting mixture was left under stirring for 2 h at 80 °C. The mixture was then thermally aged for 24 h at 100 °C. The separation of the synthesized material from the aqueous medium was performed via filtration. It was then washed once with ethanol, three times with deionized water, and was left to dry for 3 days. The removal of the organic phase (CTAB) was achieved by calcination in an oxidative atmosphere for 6 h at 550 °C (heating rate of 1 °C·min^−1^). The molar ratios of the reactants are presented in Table 1.

#### 2.1.2. Mixing MTA with Ca-Doped and Ca/Ce Co-Doped MSNs at Different Ratios

MTA powder (MTA+, Cerkamed, Stalowa Wola, Poland) was hand-mixed in a mortar with Ca/Ce co-doped and Ca-doped MSNs at 3 ratios (30.70, 50.50, and 70.30), resulting in 6 testing powder groups: CeMTA 30.70, CeMTA 50.50, CeMTA 70.30, CaMTA 30.70, CaMTA 50.50, and CaMTA 70.30. Three disc-shaped specimens for each powder group (width: 4.8 mm, height: 1.8 mm) were also prepared by mixing each powder with distilled water.

### 2.2. Material Characterization

#### 2.2.1. Fourier-Transform Infrared Spectroscopy (FT-IR)

Fourier-transform infrared spectroscopy (FTIR) was performed using a Spectrum 1000 (Perkin-Elmer, Waltham, MA, USA) spectrometer. The samples were ground with potassium bromide (KBr) at a ratio of 1:100 (samples to KBr), and each mixture was molded into a tablet using a hydraulic press. The measurements were then conducted by collecting 10 scans within the range of 450–4000 cm^−1^.

#### 2.2.2. X-ray Diffraction Analysis (XRD)

The crystal structure and phases were identified through X-ray powder diffraction analysis, which utilized both small- and wide-angle X-ray analysis. The Miniflex II XRD (manufactured by Rigaku Co. in Tokyo, Japan) diffractometer was employed, using Bragg–Brentano θ–2θ geometry and Cu Ka radiation (λCuKa = 0.15405 nm). For the small-angle X-ray diffraction, the scanning range covered 1.5–10°, with a step size of 0.02° and a step time of 3.6 s, while for the wide-angle X-ray diffraction, the scanning range was 5–75°, with a step size of 0.02° and a step time of 1 s.

#### 2.2.3. Scanning Electron Microscopy (SEM)

The analysis of the morphology of the synthesized powders was conducted using a field-emission scanning electron microscopy system (JEOL JSM-7610F Plus) supported by an Oxford AZTEC ENERGY ADVANCED X-act energy-dispersive X-ray spectroscopy (EDS) system (JEOL Ltd., Tokyo, Japan).

### 2.3. Biological Evaluation of Materials

#### 2.3.1. Isolation of Human Gingival Fibroblasts (hGFs)

Primary human fibroblasts cell lines were established from human gingival biopsies of healthy donors. Pieces of mucosa were detached during third molar routine extractions. The enzymatic separation technique was used. The institutional ethical committee approved the protocol (111/01-02-2021), and patients signed their informed consent forms prior to the extraction procedures. Cell cultures were developed in flasks with 5 mL of DMEM supplemented with 10% fetal bovine serum (FBS, Invitrogen, ThermoFisher Scientific, Waltham, MA, USA) and antibiotics (100 U/mL medium of penicillin, 100 mg/mL streptomycin, Invitrogen) (1% PS). The monitoring of cell growth was conducted with an optical microscope. When 80% confluence was achieved, fibroblasts were detached via trypsinization (using 0.25% trypsin/1 mM EDTA) and then subcultured in 75 mL flasks at 37 °C in an incubator in an air atmosphere with 95% humidity and 5% CO_2_. Trypsinization was performed when cells developed at 80% of the flask and the passage of cell cultures was performed. Passage 5 was used during the experiments of this in vitro study. More specifically, the primary cell culture was developed through enzymatic procedures. During routine third molar extractions, soft tissue from gingival was cut and sterilized using antibiotics. This tissue was minced into small pieces and then placed into an Eppendorf-containing enzymatic solution [Dispase II and Collagenase I]. This procedure was repeated until all the pieces of soft tissue could pass through the tip of a 1 mL pipette. Centrifugation at 850 rpm followed, supernatant liquid was removed, and cells were re-suspended in cell culture medium [DMEM] supplemented with antibiotics [1%PS] and serum [10%]. The cells were cultured in a 25 mL flask. The procedure is presented in Supplementary File S1 (Figure S1).

#### 2.3.2. Cell Viability Evaluation—Indirect Experiments

For the indirect experiments, the materials’ eluates were prepared from each material and MSNs (CeMTA 30.70, CeMTA 50.50, CeMTA 70.30, CaMTA30.70, CaMTA 50.50, CaMTA70.30, MTA, Ca/Ce co-doped MSNs, Ca-doped MSNs) by performing a series of dilutions in DMEM (250, 125, and 60 μg/mL). HGFs were seeded in 96-well plates (2 × 10^4^ cells/well) and were left for 24 h to attach in a 5% CO_2_ incubator at 37 °C and 100% humidity. Prior to contact with hGFs, all diluted materials were disinfected with UV light for 90 min. Each diluted material was added to cells in triplicates and then incubated for 24, 48, and 72 h. Undoped MSN served as a negative control, whereas DMEM+penicillin/streptomycin (PS) solution served as a positive control. Mitochondrial dehydrogenase activity and cell viability were evaluated with an MTT [3-(4,5-dimethylthiazol-2-yl)-2,5-diphenyltetrazolium bromide] assay. The formation of blue formazan crystals following the addition of yellow tetrazolium salt was dissolved by DMSO, and the absorbance was then measured at a wavelength of 545 nm and a reference filter of 630 nm via a microplate reader (ThermoFisher Scientific, Waltham, MA, USA). These experiments were performed twice.

#### 2.3.3. Total Antioxidant Capacity (TAC)

For the evaluation of the antioxidants, 2 well plates corresponding to 24 and 72 h were used for the corresponding materials (CeMTA 30.70, CeMTA 50.50, CeMTA 70.30, CaMTA30.70, CaMTA 50.50, CaMTA70.30, MTA, Ca/Ce co-doped MSNs, Ca-doped MSNs).

Prior to contact with hGFs, all disc specimens were disinfected with UV light for 90 min. HGFs were then added to the wells (2 × 10^4^ cells/well) and were incubated for 24, 48, and 72 h. At each time point, the medium containing DMEM and hGFs was removed and used for the measurement of total antioxidant capacity, while disc specimens along with the attached hGFs were left in the wells and were prepared for the dehydration fixation process.

The total antioxidant capacity was measured using Cayman’s antioxidant assay (709001; Cayman Chemical Company, Ann Arbor, MI, USA). Moreover, 10 μL of each sample corresponding to 24, 48, and 72 h in duplicates, as well as TROLOX preparations, were placed in a 96-well plate. Furthermore, 10 μL of metmyoglobin, 150 μL of chromogen, and 40 μL of H_2_O_2_ were then added to each sample. Incubation for 5 min was performed and the absorbance at 405 nm was monitored using a microplate reader (Thermo Scientific Multiskan FC, Waltham, MA, USA).

#### 2.3.4. Dehydration Fixation—Scanning Electron Microscopy (SEM)

Moreover, 10%, 30%, 50%, 70%, 90%, and 100% ethanol concentrations with distilled water were prepared. Furthermore, 10% ethanol was added to the wells containing disc specimens for 10 min and was then replaced by 30% ethanol. The same procedure was performed with all ethanol concentrations. The addition of 100% ethanol was performed twice. Following the removal of ethanol, hexamethyldisilazane (HMDS) was added and left for 20 min. The specimens were observed using a field-emission scanning electron microscopy system (JEOL JSM-7610F Plus) supported by an Oxford AZTEC ENERGY ADVANCED X-act energy-dispersive X-ray spectroscopy (EDS) system (JEOL Ltd., Tokyo, Japan).

#### 2.3.5. Statistical Analysis

Statistical analysis for all experiments was performed using the paired sample *t*-test, and the level of statistical significance was set at 0.05 (*p* < 0.05).

## 3. Results

### 3.1. Material Characterization

A complete characterization of the MSNs used in this study is presented in our previous publication (16). In brief, the sol–gel technique was successfully utilized for the development of highly bioactive Ca-doped and Ca/Ce co-doped mesoporous nanopowders. The SEM analysis revealed distinct morphologies with some degree of aggregation. Small-angle XRD suggested partial disorder in the hexagonal pore arrangement of doped nanoparticles. N_2_ porosimetry indicated mesoporous characteristics in Si-based nanomaterials and potential pore blocking in their doped counterparts, with variations in specific surface area and pore diameter linked to doping levels. The Ca-doped MSNs presented a high surface area of around 650 m^2^/g, 4.1 nm pore diameter, and 5.24 cc/g pore volume, while the Ca/Ce co-doped MSNs presented a surface area of around 495 m^2^/g, 3.9 nm pore diameter, and 2.69 cc/g pore volume.

#### 3.1.1. Fourier-Transform Infrared Spectroscopy (FT-IR)

**Figure 1:**
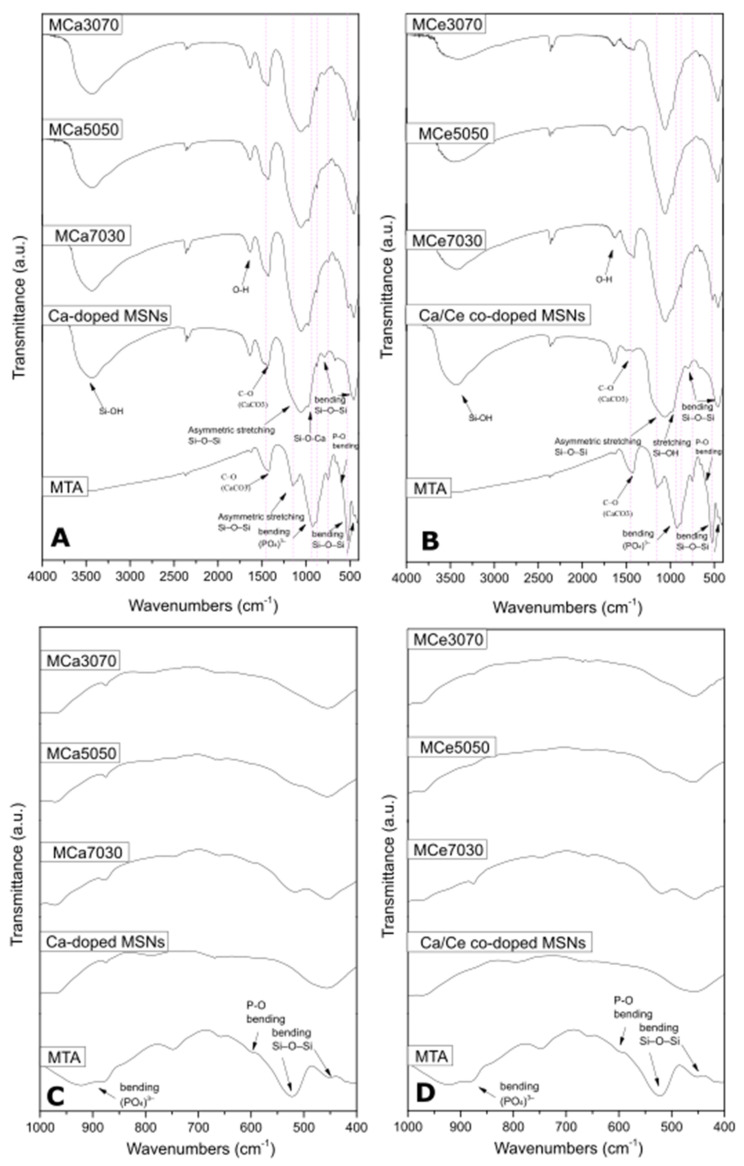
FTIR spectra of all materials. (**A**) FTIR spectra of Ca-doped MSNs and their powder samples with MTA. (**B**) FTIR spectra of Ca/Ce co-doped MSNs and their powder samples with MTA. (**C**) Selected spectra in the region 400–1000 cm^−1^ of (**A**). (**D**) Selected spectra in the region 400–1000 cm^−1^ of (**B**).

The FTIR spectra of all powder groups are presented in Figure 1. The composition of MTA+ powder includes calcium oxide, oxides of silicon, iron, aluminum, sodium, potassium, bismuth, magnesium, zirconium, and calcium phosphate. The vibration of the phosphate group is quite weak in that region. Nevertheless, the presence of a shoulder at 600 cm^−1^ confirms the presence of phosphates in MTA. However, probably due to the overlapping of the characteristic phosphate peak at 550 cm^−1^ with the peak at 515 cm^−1^ attributed to the bending vibration of the Si-O-Si bond, the peak at 550 cm^−1^ cannot appear while a shifting of the peak from 515 cm^−1^ to 522 cm^−1^ is observed. The broad band between 3100 and 3654 cm^−1^ in all MSNs powder groups corresponds to the hydroxyl groups of absorbed water. This band is less intense in the synthesized powders containing Ca/Ce co-doped MSNs. The shoulder at 972 cm^−1^ in the Ca/Ce co-doped MSNs powder groups is correlated to the stretching vibration of Si–OH bonds, whereas in the Ca-doped MSNs powder groups, the shoulder at the same wavelength is correlated to Si-O-Ca bond vibrations. Asymmetric stretching vibrations of Si-O-Si bonds are present between 984 and 1100 cm^−1^, while Si-O-Si bending vibrations are present between 458 and 796 cm^−1^. The peaks around 1428 and 1470 cm^−1^ are attributed to the C-O bond vibrations of CaCO_3_ which are more intense in MTA and Ca-doped MSNs powder groups due to the presence of more calcium ions [16,25,26,27].

#### 3.1.2. X-ray Diffraction Analysis (XRD)

**Figure 2:**
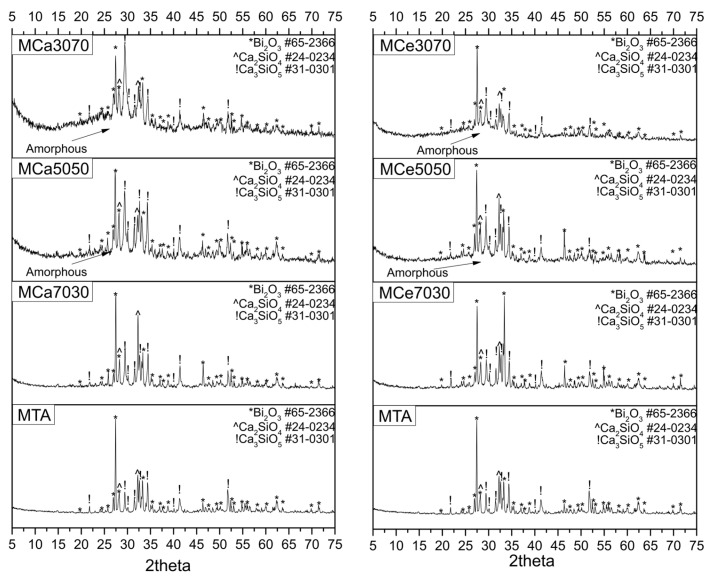
XRD diffractograms of all powder samples with MTA.

The XRD patterns, which are presented in Figure 2, confirmed the amorphous nature of the powder materials containing MSNs. This amorphous phase increased with the increase in MSNs concentration in the material. The presence of bismuth oxide was the highest (69%) in MTA samples, while a decrease in its presence was noticed with the decrease in MTA concentration, with CaMTA70.30 showing the lowest percentage (33%). The amount of calcium silicate varied between 7% and 14%, with MTA having the highest percentage, and the highest percentage of tricalcium silicate was detected in CaMTA70.30 (36%). In our study, X-ray powder diffraction (XRD) was employed to analyze the crystal structure of the synthesized nanoparticles. To determine the proportion of amorphous and crystalline phases, software tools like Jade 6 and PeakFitWin 1 were utilized. Jade software aided in identifying the crystal structure and phases contributing to the diffraction pattern by comparing the observed peaks with known patterns in its database (PDF cards). Then, using PeakFitWin, a peak fitting analysis was performed to quantify the relative proportions of each phase in the sample, allowing the calculation of the area under each peak and thus determining the proportion of each phase (Table 2).

#### 3.1.3. Scanning Electron Microscopy (SEM)

**Figure 3:**
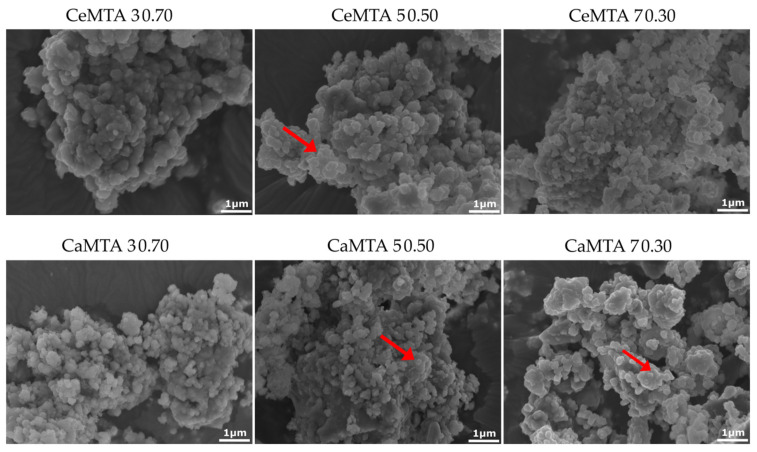
SEM images of the prepared powder samples with MTA. Magnification ×15,000, bar 1 μm.

Mesoporous materials presented a characteristic morphology as presented at SEM microphotographs. The size of the nanoparticles were at scale while aggregates were shown. The SEM microphotographs/images revealed that MSNs’ samples incubation in cell culture medium led to surface alterations. Aggregations of mesoporous nanoparticles were obvious [pointed out with red arrows], rendering the samples’ surface more uniform and smooth without the typical morphology of mesoporous nanoparticles [Figure 3].

**Figure 4:**
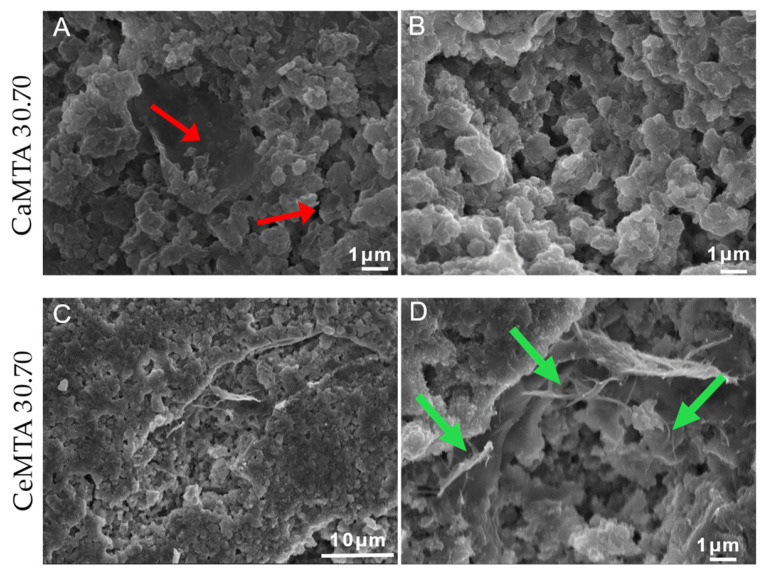
Representative images of human gingival fibroblasts after incubation with representative powder samples with MTA (CeMTA 30.70 and CaMTA 30.70). (**A**,**B**) Magnification ×10,000, bar 1 μm, (**C**) magnification ×10,000, bar 10 μm, (**D**) magnification ×8000, bar 1 μm.

Although the identification of cells was difficult due to the aggregates, they were located on the surface [pointed out with red arrows], and in higher magnifications, they showed a typical elongated morphology. At the same time, they seemed to be attached on the surface with characteristic pseudopodia [pointed out with green arrows] [Figure 4].

### 3.2. Biological Evaluation of Materials

#### 3.2.1. Cell Viability Evaluation—Indirect Experiments

**Figure 5:**
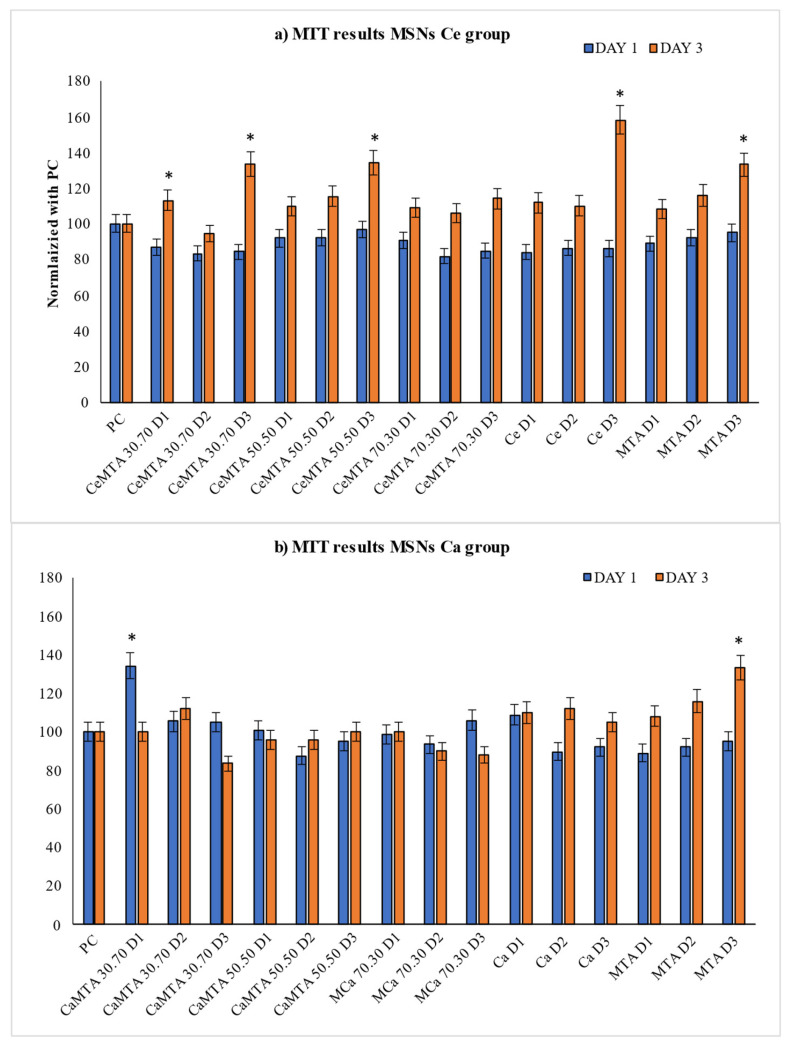
MTT results of cell viability at different concentrations of materials [μg/mL (D1 250 μg/mL, D2 125 μg/mL, D3 60 μg/mL)]. * indicates statistically significant differences (*p* < 0.05) of cell viability among the materials and the untreated cells (positive control-PC).

The cytotoxicity results are presented in Figure 5. All groups of materials were incubated for 1 and 3 days accordingly. The statistical analysis showed considerable variations among the groups. In most of the cases, all the tested materials presented non-cytotoxic behavior. Moreover, cell viability revealed an increase on day 3. Cell cultures with Ca/Ce co-doped MSNs mixed with MTA under the ratios CeMTA 30.70 and CeMTA 50.50 showed the most prominent and statistically significant increase (*p* < 0.05). For the cells treated with the MTA and Ca-doped mesoporous nanoparticles, no statistically significant alterations were recorded.

#### 3.2.2. Total Antioxidant Capacity (TAC)

**Figure 6:**
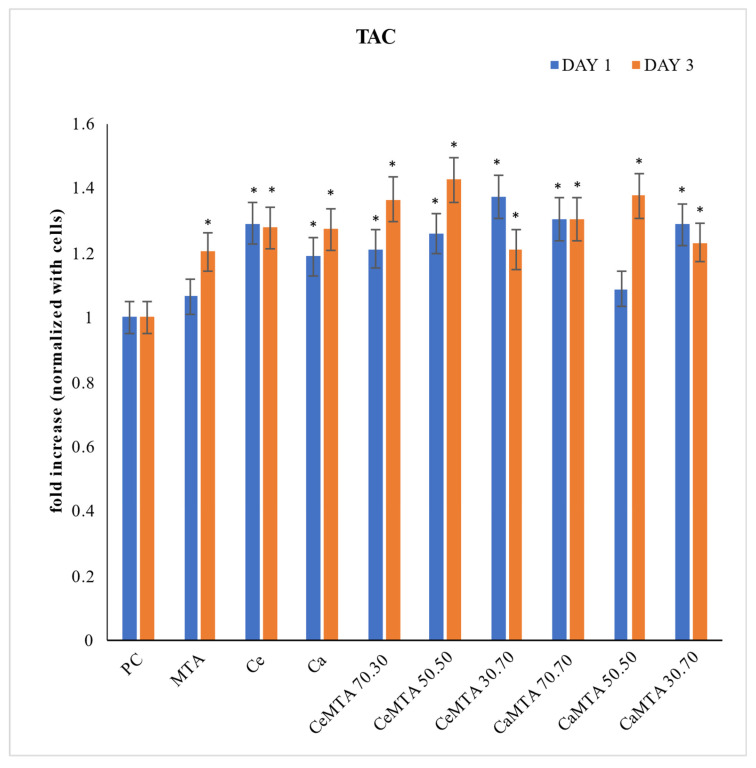
Total antioxidant capacity (TAC) assay of hGFs after culture with the tested materials. The results are expressed in mM and presented in fold modifications compared to control cells (without MSNs incubation). * indicates statistically significant differences (*p* < 0.05) of antioxidant activity among the materials and the cells (positive control-PC).

Figure 6 presents the total antioxidant capacity (TAC) of the human gingival fibroblasts (hGFs) after the incubation with the tested materials. The presence of MTA with the hGFs did not negatively affect the antioxidant capacity of the cells. The addition of the nanoparticles was found to boost the antioxidant capability of the cells (*p* < 0.05) statistically significantly. In more detail, Ca-doped MSNs showed a similar tendency to Ca/Ce co-doped MSNs in terms of increasing the antioxidant properties of the cells (*p* < 0.05). The capacity of the cells to produce antioxidants was also positively impacted by the combination of MTA and nanoparticles, especially after three days of incubation for the groups with Ca/Ce co-doped MSNs (CeMTA 70.30, CeMTA 50.50).

## 4. Discussion

MTA has provided several possibilities for pulpal therapy in both primary and young permanent teeth and has been widely applied in daily clinical practice [28]. In a systematic review and meta-analysis on vital pulp therapies in primary teeth, MTA had 89%, 89.6%, and 92.2% overall success rates in pulpotomies when compared to calcium hydroxide (46%), formocresol (85%), and ferric sulfate (79.3%), respectively [29]. MTA was also found to be superior to calcium hydroxide in indirect pulp capping in primary molars and direct pulp capping in permanent teeth [30,31]. MTA’s drawbacks such as long setting time, poor handling properties, and tooth discoloration have led researchers to the modification of MTA in order to improve its properties [32,33]. To overcome discoloration, manufacturers have replaced bismuth oxide with other stain-free radiopacifiers such as tantalum oxide which is included in NeoMTA Plus^®^ (Nusmile Inc., Houston, TX, USA) and its successor NeoMTA^®^ 2 [34]. MTAs in a stain-free premixed ready-to-use form, such as NeoPUTY^®^ (NuSmile Inc, Houston, TX, USA), have also been manufactured and have shown to be convenient in clinical practice due to their homogenous consistency and easy maneuverability in addition to avoiding mixing errors and decreasing waste of the material [34,35]. Another alternative to traditional MTA that has been lately used is Biodentine (Septodont, Saint-Maur-des-Fossés, France) which is considered a dentine substitute with the same endodontic indications as MTA [33,36]. Biodentine also contains tricalcium silicate; however, its particle size provides a more dense and less porous structure compared to MTA [37]. It has a reduced setting time and improved physical, mechanical, and handling properties [38,39]. Less tooth discoloration has been reported in teeth treated with Biodentine due to the presence of zirconium oxide as a radiopacifier instead of bismuth oxide which is found in MTA [39,40]. Therefore, the improvement in MTA properties could enhance its clinical performance.

Nanotechnology has gained extensive interest in the field of medicine and has been utilized in several aspects such as diagnosis, monitoring, operating equipment, vaccine development, drug delivery, and regenerative medicine [41]. Drug delivery is one of the most promising aspects of nanotechnology, where nanoparticles can act as carriers that deliver drugs to specific body cells or tissues. Nanoparticles have favorable surface properties allowing them to target diseased cells and avoid healthy cells, thus increasing drug efficiency while reducing side effects. In addition, controlled drug release can be achieved with nanoparticles in order to sustain drug delivery over time [42]. Mesoporous silica nanoparticles (MSNs) have been widely studied and are known for having one of the most efficient drug delivery systems [9,43]. They have unique physicochemical properties and structural properties such as adjustable pore size, particle size, high surface area, and pore volume, as well as the ability to undergo surface modification [9]. MSNs are used in various biomedical applications such as anticancer therapy, infectious diseases, and bone disorders. They are also used for diagnostic purposes by loading them with dyes and contrast agents [44]. In anticancer therapy, where the goal is targeting cancerous cells with minimal damage to healthy cells, different modifications of MSNs have been explored [45]. One example is the use of MSNs for the delivery of paclitaxel, which is a hydrophobic anticancer drug; MSNs were found to improve its solubility and thus its cytotoxicity against liver carcinoma cells [46]. Another example is its application in photodynamic therapy where a photosensitizer is targeted with a specific light wavelength, generating reactive oxygen species (ROS) that destroy nearby cancerous cells [45]. The addition of MSNs to photosensitizers was found to improve their anticancer properties by rapidly producing ROS after irradiation [47]. MSNs have also been applied in the field of dentistry. They have shown great potential in dental restorative materials. Adding chlorhexidine encapsulated into MSNs to glass ionomer cement or dental resin boosted the material’s antibiofilm and antibacterial properties through the controlled release of chlorhexidine over a long time, without affecting their mechanical properties [21,22]. Calcium-doped MSNs incorporated into dental resin led to improvements in the mechanical properties of the resin, the induction of apatite mineralization, and the inhibition of bacterial growth [48]. MSNs’ effect on dental hypersensitivity has also been studied. Nanohydroxyapatite (nHAp) was found to effectively occlude dentinal tubules by facilitating crystal formation and deposition in demineralized teeth [49]. Also, the addition of epigallocatechin-3-gallate (EGCG), which has antibiofilm and anti-inflammatory properties, into nHAp MSNs not only showed dentinal tubule occlusion due to the presence of nanohydroxyapatite but also a significant inhibition of S.mutans biofilm formation and growth on the surface of dentin due to the continuous release of EGCG [50]. Several studies have evaluated the addition of nanoparticles to MTA. Somaie et al. (2023) modified MTA with the addition of nanographene oxide, and the results showed that the addition of 1 wt% nanographene oxide can significantly improve the setting time and antibacterial activity without compromising the compressive strength of MTA [51]. Hernandez-Delgadillo et al. (2017) supplemented MTA with bismuth lipophilic nanoparticles which exhibited positive effects on the antimicrobial and antibiofilm activities of MTA without affecting its mechanical properties [52]. Silver nanoparticles as additives to MTA have also been investigated in the literature. These nanoparticles were found to enhance the antimicrobial properties of MTA as well as its push-out bond strength and compressive strength [53].

In the current study, the modification of MTA with the addition of cerium- and calcium-doped mesoporous silica nanoparticles was evaluated. Following the synthesis of the materials, a characterization was performed. FTIR and XRD results confirmed the successful modification of MTA with MSNs, while SEM images confirmed the presence of MSNs and showed aggregations of non-uniform shape. Cell viability/proliferation analysis was performed using the MTT assay. The results showed a significant enhancement in cell viability/proliferation in the CeMTA groups. On day three, a significant enhancement was recorded, especially in the 60 μg/mL dilution and in the CeMTA 50.50 and CeMTA 30.70 groups. This comes in agreement with a study by Tsamesidis et al. (2021) where a significant positive effect of artemisinin loaded with cerium-doped mesoporous silica nanoparticles on cell viability was recorded on day three. In their study, however, 125 μg/mL dilution was regarded as the most beneficial [16]. In the CaMTA groups, a significant enhancement in cell viability was recorded on day one in the CaMTA 30.70 group with a dilution of 250 μg/mL; however, a major drop in cell viability was recorded on day three. A slight enhancement in cell viability was mostly shown in the 125 μg/mL dilutions and in the groups CaMTA 30.70 and CaMTA 50.50. The increase in the calcium-doped MSNs’ ratio (CaMTA 70.30) showed a slight enhancement on day three in the 250 μg/mL dilution and a decrease in cell viability on day three in the 125 and 60 μg/mL dilutions. By comparing the results of both categories CeMTA and CaMTA, loading MTA with Ca/Ce co-doped mesoporous silica nanoparticles was recorded as most beneficial to the viability of hGFs and could therefore be considered as an alternative to traditional MTA. Jun et al. (2019) also demonstrated that loading MTA with cerium nanoparticles promoted a significant increase (90%) in the viability of human dental pulp stem cells (hDPSCs), leading to odontoblastic differentiation [24]. It is well known that calcium ions are important in cellular division processes [54,55]. They play a fundamental role in activating or inhibiting many intracellular reactions that control cell cycles, especially mitosis. In particular, the increase in the concentration of calcium ions guides cells through mitosis via different mechanisms such as nuclear envelope breakdown, chromosome condensation, spindle fiber formation, and sister chromatid separation [54]. In MTA, calcium ions are present as calcium oxide which reacts with tissue fluids, forming calcium hydroxide [56]. Studies that have evaluated the effect of increasing the concentration of calcium ions in MTA have found that there is a limit to the effect of calcium ions on cellular mineralization [57,58]. High concentrations of calcium ions could result in a cytotoxic effect trigger, resulting in cellular apoptosis and/or necrosis [57]. This might explain the drop in cell viability on day 3 in the CaMTA 70.30 group in our study. In contrast, the beneficial role of Ce ions in cell viability, proliferation, and differentiation has been demonstrated. More specifically, Mahaparta C. et al. (2016) reported that in cells CeNMs-treated under oxidative stress [ROS], DNA double-stranded splits and breakdowns were prevented, lipids concentrations remained stable, and proteins were rescued from oxidation. Ce ions are reported to have a protective role under oxidative conditions, repairing DNA damage and rescuing lipid peroxidation and protein oxidation. These characteristics render them a promising material in tissue engineering [59].

An evaluation of the materials’ total antioxidant capacity was performed using an antioxidant assay. The presence of MSNs with MTA was found to boost the antioxidant capacity of hGFs on day three of incubation in all groups, except the CeMTA 30.70 and CaMTA 30.70 where antioxidant capacity decreased. The 50.50 ratio in both CeMTA and CaMTA groups showed the highest increase in antioxidant capacity with time, while the 70.30 ratio was found to be more effective in the CeMTA group, whereas in the CaMTA group, no change was noticed on day three. These results show that the modification of MTA with equal or higher levels of Ca/Ce co-doped MSNs positively affects the antioxidant capacity of the material. Cerium is well known in the literature as an antioxidant [24,60]. Reactive oxygen species (ROS) induce oxidative stress causing adverse effects on cells and tissues; therefore, the reduction in excessive ROS is necessary to maintain healthy biological functions [20]. In healthy cells, cerium nanoparticles act as antioxidants by scavenging ROS, while in pathogens, they act as a prooxidant by generating ROS and thus cell damage [61]. Pinna et al. (2021) found that doping MSNs with cerium resulted in a higher ROS scavenging capability and produced favorable osteogenic and anti-osteoclastogenic properties compared to undoped MSNs [62]. Also, Jun et al. (2019) proved in their study that cerium nanoparticles in MTA provided a therapeutic response through the downregulation of intra-cellular ROS during the odontoblastic differentiation of hDPSCs [24]. More specifically, they supported that Ce nanoparticles enhance cell survivability against oxidative stress [H_2_O_2_], a condition referred at the early stage of inflammation. Moreover, Ce nanoparticles were reported to not only to promote cell proliferation but to cause an upregulation of cell differentiation, too. That is why they are suggested in applications for tissue engineering. Based on the MTT assay results of our study, the modification of MTA with Ca/Ce co-doped MSNs increased cell viability compared to the control group (MTA), while the modification of MTA with Ca-doped MSNs provided comparable mitochondrial activity/cell viability with the control group (MTA). Therefore, Ca/Ce co-doped MTA could be considered as a promising candidate in vital pulp therapy treatment. The limitation of this study is the absence of an antibacterial assessment of the studied materials. Additional studies are needed to demonstrate the antibacterial and mechanical properties of the developed mixtures, as well as the setting time of MTA, and the potential of further modifying MSNs with other ions or drugs that could further improve the properties of MTA.

## 5. Conclusions

1. All tested materials proved to be biocompatible with the specific cells.
2. The most beneficial biological behavior was reported for CeMTA 50.50 concentration.
3. The modification of MTA with Ca-doped MSNs proved to be biocompatible without presenting beneficial biological behavior compared to traditional MTA.
4. The modification of MTA with Ca/Ce co-doped MSNs could promote mitochondrial activity and thus could be used as a promising alternative to traditional MTA in vital pulp therapy treatment.

The tested material can be used with low risk, as evidenced by the increased cell proliferation of the utilized human gingival fibroblasts primary cell line. To further elucidate their potential positive effect on cell metabolic pathways, more tests are needed. Further investigation concerning the antibacterial properties of modified MTA with Ca/Ce co-doped MSNs would open a new treatment modality in infectious conditions in the oral cavity.

## Figures and Tables

**Table 1:** Reaction stoichiometry for the produced MSNs.

Sample	Molar Ratio of Reactants
Ca-doped MSNs	0.6 TEOS/0.13 CTAB/0.4 NaOH/1240 H_2_O/0.4 Ca
Ca/Ce co-doped MSNs	0.6 TEOS/0.13 CTAB/0.4 NaOH/1240 H_2_O/0.375 Ca/0.025 Ce

**Table 2:** Quantification of XRD results.

Sample	Amorphous	Bi_2_O_3_ #65-2366	Ca_2_SiO_4_ #24-0234	Ca_3_SiO_5_ #31-0301	
MTA		69%	14%	17%	100%
CaMTA 30.70	7%	65%	12%	16%	100%
CaMTA 50.50	15%	61%	8%	16%	100%
CaMTA 70.30	24%	33%	7%	36%	100%
MTA		69%	14%	17%	100%
CeMTA 30.70	6%	68%	11%	15%	100%
CeMTA 50.50	14%	64%	9%	13%	100%
CeMTA 70.30	17%	63%	8%	12%	100%

## Data Availability

Data are contained within the article.

## Supplementary Materials

The following supporting information can be downloaded at: https://www.mdpi.com/article/10.3390/cimb46040188/s1, Figure S1: Schematic representation of primary cell line of human gingival fibroblasts establishment.
