# Supplementary material for: Effect of Modified Bioceramic Mineral Trioxide Aggregate Cement with Mesoporous Nanoparticles on Human Gingival Fibroblasts
Source: Curr Issues Mol Biol. 2024 Mar 30;46(4):3005–21. doi: 10.3390/cimb46040188 (PMC11048828; doi:10.3390/cimb46040188)
Supplement: Supplementary file 1 [file cimb-46-00188-s001.zip › cimb-2891149-supplementary.pdf]

Supplementary file 1.

Primary cell culture was developed through the enzymatic dissociation method. During routine third molar extraction, soft tissue from gingival was cut and sterilized using antibiotics. This tissue was minced into small pieces and then placed into an Eppendorf containing enzymatic solution [Dispase II and Collagenase I]. This procedure was repeated until all the pieces of soft tissue could pass through the tip of 1ml pipette. Centrifugation at 850rpm followed, supernatant liquid was removed, and cells were re-suspended in cell culture medium [DMEM] supplemented with antibiotics [1%PS] and serum [10%]. Cells were cultured in a $25mm^3$ flask. The procedure is presented in supplementary figure 1. Cells were identified at the surface of flask and their position was marked to monitor their development. Initially, dispersion of cells was scarce. Cells were monitored every 3 days and by the 16th day 60% of fluency was achieved. The cells were split and put into two $75mm^3$ flasks to proliferate. The experimental procedures were performed at the Department of Basic Dental Sciences, Division of Dental Tissues Pathology and Therapeutics, School of Dentistry, Faculty of Health Sciences, Aristotle University of Thessaloniki, Greece.

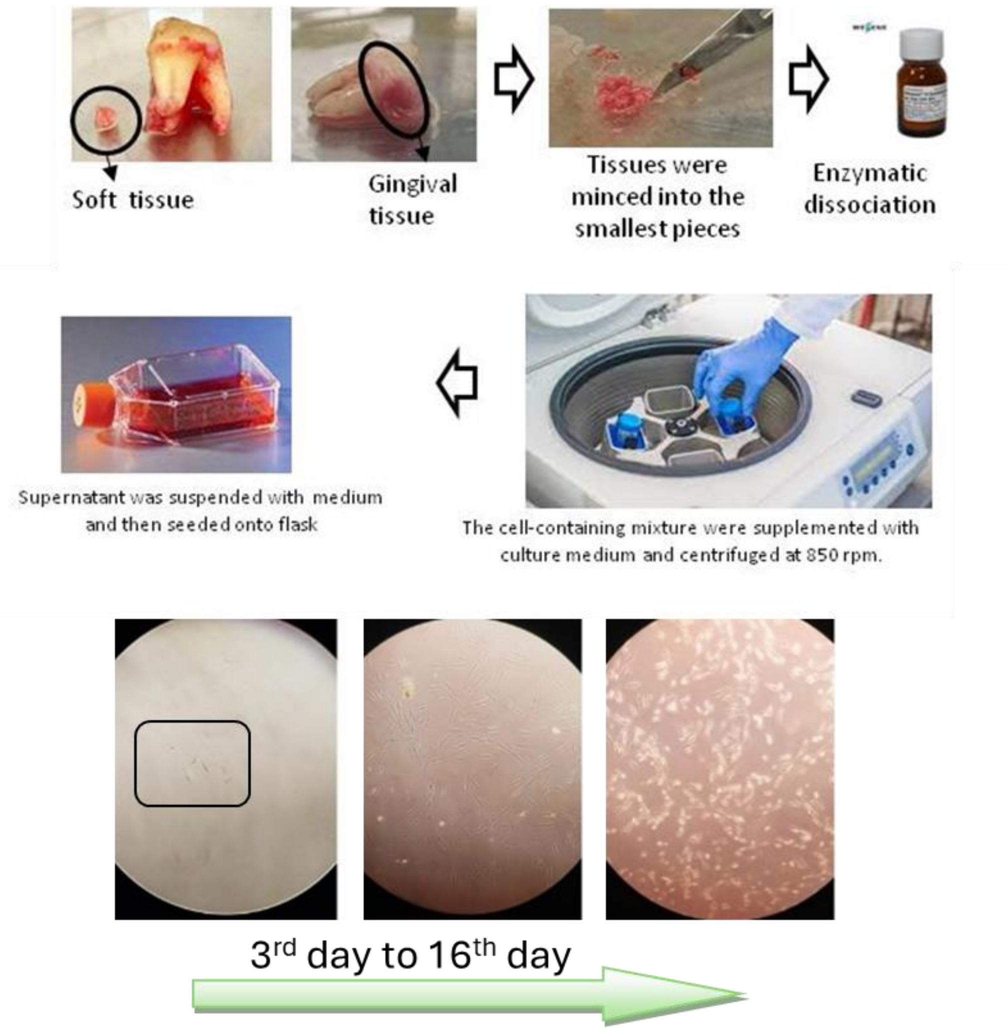

Supplementary figure 1. Schematic representation of primary cell line of human gingiva fibroblasts establishment.
